# Supplementary material for: Content-rich biological network constructed by mining PubMed abstracts
Source: BMC Bioinformatics. 2004 Oct 8;5:147. doi: 10.1186/1471-2105-5-147 (PMC528731; doi:10.1186/1471-2105-5-147)
Supplement: Additional File 2 — The original results of the above study (non-essential files are deleted to keep the file size under the limit set by BMC bioinformatics). [file 1471-2105-5-147-S2.bz2 › chilibotAdditionalFile2/dip50/700ID10869074E18033/html/MIND_MINC.html]

 


 **MIND** and **MINC** 
  
Found 51 abstracts in PubMed, retrieved 50.  
 

 What does Google say? 
 PDF only 
| .edu only 

---

**Interactive relationship** (e.g. stimulation, inhibition, etc)

**Inhibitory relationship**- The results support a model in which  **MinD**  recruits  **MinC**  to its site of action and in which FtsZ ring assembly at each of the cell ends is blocked in an intermittent and alternate fashion.  Ref: 10515933 J Bacteriol, 1999
- Formation of the MinE ring required  **MinD**  but was independent of  **MinC**  and continued in nondividing cells in which FtsZ function was inhibited.  Ref: 9393861 Cell, 1997
- The results suggest a mechanism whereby  **MinD**  is required both to pilot  **MinC**  to the cell poles and to constitute a functional division inhibitor.  Ref: 10411726 Mol Microbiol, 1999
- This activity of min is achieved through the action of an inhibitor of division,  **MinC** , that is activated by  **MinD**  and topologically regulated by MinE.  Ref: 10540287 Mol Microbiol, 1999
- **MinC**  mediated division inhibition can also be activated in a  **MinD**  independent fashion by the DicB protein of cryptic prophage Kim.  Ref: 12003935 J Bacteriol, 2002
- New  **minC**  mutations suggest different interactions of the same region of division inhibitor  **MinC**  with proteins specific for  **minD**  and dicB coinhibition pathways.  Ref: 1729222 J Bacteriol, 1992
- Previous genetic analysis has shown that either  **MinD**  or an apparently unrelated protein, DicB, cooperates with  **MinC**  to inhibit division.  Ref: 1729222 J Bacteriol, 1992
- Accurate placement of the division septum at the midpoint of Escherichia coli cells requires the combined action of a general division inhibitor  **MinC** , a site specific suppressor of division inhibition MinE, and an ATPase  **MinD**  that is required for proper functioning of both  **MinC**  and MinE.  Ref: 10220403 Proc Natl Acad Sci U S A, 1999
- These studies suggest that  **MinD**  Ng is involved in inhibiting gonococcal cell division, likely in conjunction with  **MinC**  Ng.  Ref: 11591668 J Bacteriol, 2001
- USA 87 1129 113 990, these results provide evidence that, in addition to promoting division inhibition with  **MinD** , protein  **MinC**  acts in concert with DicB to inhibit division by a second,  **MinD**  independent process.  Ref: 2211516 J Bacteriol, 1990
- Some mutations exhibited preferential resistance to one or the other coinhibitor, suggesting that two distinct proteins, possibly  **MinD**  and DicB themselves, interact in slightly different manners with the same region of  **MinC**  to promote division inhibition.  Ref: 1729222 J Bacteriol, 1992
- Several mutations affecting the mid to C terminal parts of  **MinD**  led to a protein probably unable to interact with DivIVA, but that could still stimulate division inhibition by  **MinC** .  Ref: 11886553 Mol Microbiol, 2001
- In Escherichia coli, site selection is dependent on  **MinC** ,  **MinD**  and MINE  **MinC**  acts, with  **MinD** , to inhibit division at sites other than the midcell by directly interacting with FTSZ Here we report the crystal structure to 2.2 A of  **MinC**  from Thermotoga maritima.  Ref: 11350934 EMBO J, 2001
**Neutral relationship**- However, interactions were observed among various members of the Min system, and MinE was found to reduce the interaction between  **MinC**  and  **MinD** .  Ref: 8752322 J Bacteriol, 1996
- **MinD**  and MinE regulate  **MinC**  activity by modulating its cellular location in a unique fashion.  Ref: 11285221 EMBO J, 2001
- iii both  **MinC**  and MinE can be recruited to  **MinD** .ATP decorated vesicles.  Ref: 12533449 J Bacteriol, 2003
- Here, we present evidence that the binding of D  **MinC**  to  **MinD**  endows the  **MinC**   **MinD**  complex with a more specific affinity for a septal ring associated target in vivo.  Ref: 12003935 J Bacteriol, 2002
- Also, it is argued that binding of  **MinD**  to the membrane activates  **MinC** .  Ref: 12486056 J Bacteriol, 2003
- Analysis of  **MinC**  reveals two independent domains involved in interaction with  **MinD**  and FtsZ.  Ref: 10869074 J Bacteriol, 2000
- **MinC**  oscillates along with  **MinD**  such that the time integrated concentration of Z  **MinC**  at the membrane is minimal, and hence the stability of FtsZ polymers is maximal, at the cell center.  Ref: 12003935 J Bacteriol, 2002
- We observed that  **MinD**  dimerized in the presence of ATP and interacted with  **MinC** .  Ref: 12486056 J Bacteriol, 2003
- In the presence of a phospholipid bilayer,  **MinD**  bound to the bilayer and recruited  **MinC**  in an ATP dependent manner.  Ref: 12486056 J Bacteriol, 2003
- A conserved sequence at the C terminus of  **MinD**  is required for binding to the membrane and targeting  **MinC**  to the septum.  Ref: 12519187 Mol Microbiol, 2003
- These results suggest that MinE induces a conformational change in  **MinD**  bound to the bilayer that results in the release of  **MinC** .  Ref: 12486056 J Bacteriol, 2003
- In contrast,  **MinC**  was unable to displace MinE bound to the  **MinD**  bilayer complex.  Ref: 12486056 J Bacteriol, 2003
- The addition of  **MinD** , a peripheral membrane protein that interacts with  **MinC** , results in GFP  **MinC**  appearing on the membrane.  Ref: 10540287 Mol Microbiol, 1999
- ... the C terminal domain D  **MinC**  is required for dimerization as well as for interaction with the  **MinD**  protein.  Ref: 12003935 J Bacteriol, 2002
- Here we show that functional green fluorescent protein  **MinC**  displays a very similar oscillatory behavior which is dependent on both  **MinD**  and MinE and independent of FtsZ.  Ref: 10515933 J Bacteriol, 1999
- Targeting of D  **MinC**   **MinD**  and D  **MinC**  DicB complexes to septal rings in Escherichia coli suggests a multistep mechanism for  **MinC**  mediated destruction of nascent FtsZ rings.  Ref: 12003935 J Bacteriol, 2002
- **MinD**  plays a central role in this process, as it positions and activates  **MinC**  and is dependent on DivIVA for its own positioning at the poles.  Ref: 11886553 Mol Microbiol, 2001
- **MinC**  dynamics involves a membrane association dissociation cycle that is driven by the activities of the  **MinD**  ATPase and the MinE topological specificity factor, which themselves undergo coupled oscillatory localization cycles.  Ref: 12533449 J Bacteriol, 2003
- Thus,  **MinD**  does not merely attract  **MinC**  to the membrane but also aids  **MinC**  in specifically binding to, or in close proximity to, the substrate of its Z  **MinC**  domain.  Ref: 12003935 J Bacteriol, 2002
- By using computer simulations we show that Min protein dynamics can be described accurately by using the following assumptions i the  **MinD**  ATPase self assembles on the membrane and recruits both  **MinC** , an inhibitor of Z ring formation, and MinE, a protein required for  **MinC**   **MinD**  oscillation, ii a local accumulation of MinE is generated by a pattern formation reaction that is based on local self enhancement and a long range antagonistic effect, and iii it displaces  **MinD**  from the membrane causing its own local destabilization and shift toward higher  **MinD**  concentrations.  Ref: 11734639 Proc Natl Acad Sci U S A, 2001
- We show that cytoplasmic  **MinC**  is poised near the monomer dimer equilibrium and propose that it only becomes entirely dimeric once recruited to the membrane by  **MinD** .  Ref: 11673440 J Bacteriol, 2001
- In the presence of ATP  **MinD**  binds  **MinC**  and goes to the membrane the ATPase is not stimulated and the complex is stable.  Ref: 12675792 Mol Microbiol, 2003
- We have isolated and sequenced  **minC**  mutations that suppress division inhibition caused by overproduction of either DicB or  **MinD**  proteins.  Ref: 1729222 J Bacteriol, 1992

**Non-interactive relationship** (e.g. studied together, co-existance, homology, etc.)

- We report the cloning of the wild type allele of divIVB1 and show that the mutation lies within a stretch of DNA containing two open reading frames whose predicted products are in part homologous to the products of the Escherichia coli minicell genes  **minC**  and  **minD** .  Ref: 1400224 J Bacteriol, 1992
- In the presence of both  **MinD**  and MinE, GFP  **MinC**  oscillates rapidly between the halves of the cell.  Ref: 10540287 Mol Microbiol, 1999
- Insertional inactivation of either  **minC**  or  **minD**  or the presence of the divIVB region on plasmids produces a severe minicell phenotype in wild type cells.  Ref: 1400225 J Bacteriol, 1992
- The last two ORFs minCD are homologous to  **minC**  and  **minD**  of Escherichia coli but a minE homolog is lacking in B. subtilis.  Ref: 9765563 J Bacteriol, 1998
- BACKGROUND In Escherichia coli, the cell division site is determined by the cooperative activity of min operon products  **MinC** ,  **MinD** , and MinE.  Ref: 11566131 Structure Camb , 2001
- The joint overexpression of  **MinC**  Ng and  **MinD**  Ng from a shuttle vector resulted in a significant enlargement of gonococcal cells, while cells transformed with plasmids encoding either  **MinC**  Ng or  **MinD**  Ng alone did not display noticeable morphological changes.  Ref: 11591668 J Bacteriol, 2001
- The results also indicate that MinE stimulated dissociation of  **MinC**  from the  **MinC**   **MinD** .ATP membrane complex can, and may, occur prior to hydrolysis of the nucleotide.  Ref: 12533449 J Bacteriol, 2003
- **MinC**  activity requires that of  **MinD** , and the MinE peptide provides topological specificity by suppressing  **MinC**   **MinD**  mediated division inhibition specifically at the middle of the cell.  Ref: 10515933 J Bacteriol, 1999
- Selection of the midcell division site in Bacillus subtilis through  **MinD**  dependent polar localization and activation of  **MinC** .  Ref: 10411726 Mol Microbiol, 1999
- **MinD**  recruits  **MinC**  to the membrane, and MinE induces  **MinC**   **MinD**  to oscillate rapidly between the membrane of opposite cell halves.  Ref: 11285221 EMBO J, 2001
- With  **MinC** ,  **MinD**  forms a non specific inhibitor of division, that interacts with FtsZ.  Ref: 11248256 FEBS Lett, 2001
- Introduction of a negatively charged amino acid decreased membrane binding of  **MinD**  and its ability to activate  **MinC** .  Ref: 12867440 J Bacteriol, 2003
- **MinC**  and  **MinD**  interact to form a non specific division inhibitor that blocks septation at all potential division sites.  Ref: 10096083 Mol Microbiol, 1999
- Recruitment of  **MinC** , an inhibitor of Z ring formation, to the membrane in Escherichia coli role of  **MinD**  and MinE.  Ref: 12486056 J Bacteriol, 2003
- The activity of the promoter regions of the cell division genes ftsZ, ftsE,  **minC** ,  **minD**  and minE from Neisseria gonorrhoeae Ng was studied under different environmental conditions using lacZ translational fusions.  Ref: 11763238 Res Microbiol, 2001
- All the mutations were found to lie in  **minC**  or  **minD** , confirming the requirement of both gene products in the process of inhibition of polar sites.  Ref: 2211516 J Bacteriol, 1990
- Proper positioning of division sites in Escherichia coli requires balanced expression of  **minC** ,  **minD** , and minE gene products.  Ref: 1729222 J Bacteriol, 1992
- This positioning is achieved through a rapid oscillation of  **MinC**  from pole to pole, a process requiring  **MinD**  and MinE.  Ref: 11430835 Mol Cell, 2001
- In rod shaped bacteria such as Bacillus subtilis, division site selection is mediated by  **MinC**  and  **MinD** , which together function as a division inhibitor.  Ref: 11886553 Mol Microbiol, 2001
- This site specific inhibition of septation is mediated by the gene products of the minicell locus the minB operon that includes three genes,  **minC** ,  **minD** , and minE.  Ref: 2137246 Proc Natl Acad Sci U S A, 1990
- By overexpressing  **minC**  or  **minD** , we show that both proteins are required to block division, but that only  **MinD**  needs to be in excess of wild type levels.  Ref: 10411726 Mol Microbiol, 1999
- Division site selection in rod shaped bacteria is mediated by  **MinC**  and  **MinD** , which form a division inhibitor.  Ref: 10411726 Mol Microbiol, 1999
- In the absence of MinE, the coordinate expression of  **MinC**  and  **MinD**  leads to a general inhibition of cell division.  Ref: 1729224 J Bacteriol, 1992
- The Min system comprises  **MinD**  and  **MinC** , which form an inhibitor complex and, in Bacillus subtilis, DivIVA, which ensures that division is inhibited only in the polar regions.  Ref: 12492852 Mol Microbiol, 2003
- However, neither  **minC**  nor  **minD**  function was absolutely required for sporulation and, by implication, for asymmetric septum formation.  Ref: 8459776 Mol Microbiol, 1993
- The proper placement of the cell division site in Escherichia coli requires the site specific inactivation of potential division sites at the cell poles in a process that is mediated by the  **MinC** ,  **MinD**  and MinE proteins.  Ref: 1836760 EMBO J, 1991
- In Escherichia coli, this process depends crucially on the proteins  **MinC** ,  **MinD** , and MinE.  Ref: 11806906 Biophys J, 2002
- MinE assembles into a membrane associated ring structure near midcell and directs the localization of  **MinD**  and  **MinC**  into a membrane associated polar zone that undergoes a characteristic pole to pole oscillation cycle.  Ref: 12093736 EMBO J, 2002
- DNA sequence analysis found two open reading frames whose predicted products had significant identity to the E. coli  **MinC**  cell division inhibitor and the  **MinD**  ATPase activator of  **MinC** , and disruption of minCD function generated a minicell phenotype in B. subtilis.  Ref: 8459776 Mol Microbiol, 1993
- At normal levels of expression, both  **MinC**  and  **MinD**  are required for the division block.  Ref: 1729224 J Bacteriol, 1992
- The proper placement of the cell division site in Escherichia coli requires the site specific inactivation of potential division sites at the cell poles in a process that requires the coordinate action of the  **MinC** ,  **MinD** , and MinE proteins.  Ref: 1729224 J Bacteriol, 1992
- Accurate positioning of the division septum at the equator of Escherichia coli cells requires a rapid oscillation of  **MinD**  ATPase between the polar halves of the cell membrane, together with the division inhibitor  **MinC** , under MinE control.  Ref: 12482939 Proc Natl Acad Sci U S A, 2002
- Localization of  **MinC**  requires both early FtsZ and late PbpB division proteins, and it is completely dependent on  **MinD** .  Ref: 10411726 Mol Microbiol, 1999
- **MinD**  localizes to the cytoplasmic face of the inner membrane in Escherichia coli, and its association with the inner membrane is a prerequisite for membrane recruitment of the septation inhibitor  **MinC** .  Ref: 12424340 Proc Natl Acad Sci U S A, 2002
- The release of  **MinC**  did not require ATP hydrolysis, indicating that MinE could displace  **MinC**  from the  **MinD**  bilayer complex.  Ref: 12486056 J Bacteriol, 2003
- Similar to the results obtained with  **MinD** , we find that DicB interacts directly with D  **MinC** , that the D  **MinC**  DicB complex has a high affinity for some septal ring target s,.  Ref: 12003935 J Bacteriol, 2002
- MinE accomplishes this by imparting topological specificity to a division inhibitor coded by the  **minC**  and  **minD**  genes.  Ref: 7753804 Proc Natl Acad Sci U S A, 1995
- **MinD**  plays two roles in the MinCDE system it activates the  **MinC**  dependent division inhibition mechanism and is also required for the sensitivity of the division inhibition system to the MinE topological specificity factor.  Ref: 1729224 J Bacteriol, 1992
- **MinC**  is cytoplasmic and fails to block FtsZ assembly in the absence of  **MinD** , indicating that recruitment of  **MinC**  by  **MinD**  to the membrane enhances Z  **MinC**  function.  Ref: 12003935 J Bacteriol, 2002
- and v MinE stimulates dissociation of  **MinC**  from  **MinD** .ATP membrane complexes, even when ATP hydrolysis is blocked.  Ref: 12533449 J Bacteriol, 2003
- The results suggest a multistep mechanism for the activation of  **MinC**  mediated division inhibition by either  **MinD**  or DicB and further expand the number of properties that can be ascribed to the Min proteins.  Ref: 12003935 J Bacteriol, 2002
- When the putative ATP binding domain of  **MinD**  was altered by site directed mutagenesis, the mutant protein was no longer able to activate the  **MinC**  dependent division inhibition system.  Ref: 1836760 EMBO J, 1991
- To clarify the functional role of the bacterial cell division inhibitor  **MinD**   [ **MIND** ] , which is a membrane associated ATPase that works as an activator of  **MinC** , we determined the crystal structure of a Pyrococcus furiosus  **MinD**  homologue complexed with a substrate analogue, AMPPCP, and with the product ADP at resolutions of 2.7 and 2.0 A, respectively.  Ref: 11296216 EMBO J, 2001
- Just upstream of  **minC**  and  **minD** , and in the same orientation, are three genes whose products are homologous to the products of the E. coli shape determining genes mreB, mreC, and mreD.  Ref: 1400224 J Bacteriol, 1992
- In Escherichia coli, selection of the correct mid cell site is mediated by the  **MinC** ,  **MinD**  and MinE proteins.  Ref: 11296216 EMBO J, 2001
- Addition of MinE to the MinCD bilayer complex resulted in release of both  **MinC**  and  **MinD** .  Ref: 12486056 J Bacteriol, 2003
- This finding correlates well with the previously observed oscillatory movement of  **MinC**  and  **MinD**  in short and long cells.  Ref: 10931285 Mol Microbiol, 2000
- Minicell forming mutants of Escherichia coli suppression of both DicB and  **MinD**  dependent division inhibition by inactivation of the  **minC**  gene product.  Ref: 2211516 J Bacteriol, 1990
- Correct placement of the division septum in Escherichia coli requires the co ordinated action of three proteins,  **MinC** ,  **MinD**  and MinE.  Ref: 10096083 Mol Microbiol, 1999
- DicB shows little homology to  **MinD** , and how it stimulates  **MinC**  function has been unclear.  Ref: 12003935 J Bacteriol, 2002
- The trans acting factor is the product of the sdiA gene, which was isolated on the basis of its ability to suppress the division inhibitory effect of the  **MinC**   **MinD**  division inhibitor.  Ref: 1915297 EMBO J, 1991
- We show here that, when expressed at high levels,  **MinC**  acts as a division inhibitor even in the absence of  **MinD** .  Ref: 1729224 J Bacteriol, 1992
- Recent studies have shown that MinE, previously thought to form a static ring near the division site at the midcell position, actually joins  **MinC**  and  **MinD**  in their rapid oscillation between the cell poles.  Ref: 11378404 Curr Biol, 2001
- These results demonstrate that  **MinC**  consists of two independently functioning domains an N terminal domain capable of inhibiting FtsZ assembly and a C terminal domain responsible for localization of  **MinC**  through interaction with  **MinD** .  Ref: 10869074 J Bacteriol, 2000
- The division inhibition that results from  **MinC**  overexpression in the absence of  **MinD**  is insensitive to the MinE topological specificity factor.  Ref: 1729224 J Bacteriol, 1992
- Correct positioning of the division septum in Escherichia coli depends on the coordinated action of the  **MinC** ,  **MinD**  and MinE proteins.  Ref: 11062554 Nat Struct Biol, 2000
- Topological regulation of cell division in Escherichia coli involves rapid pole to pole oscillation of the division inhibitor  **MinC**  under the control of  **MinD**  and MinE.  Ref: 10540287 Mol Microbiol, 1999
- Roles of  **MinC**  and  **MinD**  in the site specific septation block mediated by the MinCDE system of Escherichia coli.  Ref: 1729224 J Bacteriol, 1992
- The second gene cluster contains two ORFs minCD homologous to  **minC**  and  **minD**  of E. coli but lacks a minE homolog.  Ref: 1400225 J Bacteriol, 1992
- Positioning of the midcell division plane within the bacterium E. coli is controlled by the min system of proteins  **MinC** ,  **MinD** , and MinE.  Ref: 11800919 Phys Rev Lett, 2001
- In extension of previous nucleotide sequence analysis, our results show that the order of genes in the vicinity of spoIIB is valS folC comC spoIIB orfA orfB mreB mreC mreD  **minC**   **minD**  spoIVFA spoIVFB L20 orfX L24 spoOB obg pheB pheA.  Ref: 8419299 J Bacteriol, 1993
- Selection of the correct division site at midcell is controlled by three proteins  **MinC** ,  **MinD** , and MinE.  Ref: 10633093 J Bacteriol, 2000
- Cell division inhibitors SulA and  **MinC**   **MinD**  block septum formation at different steps in the assembly of the Escherichia coli division machinery.  Ref: 10931335 Mol Microbiol, 2000
- The Min system of Escherichia coli directs cell division to the mid cell by a mechanism that involves the dynamic localization of all of its three constituent proteins,  **MinC** ,  **MinD**  and MinE.  Ref: 12368455 Microbiology, 2002
